# Challenges and Strategies in Medication Management for Patients With Multiple Comorbidities

**DOI:** 10.7759/cureus.85992

**Published:** 2025-06-14

**Authors:** Ayomide H Adeyemi, Bernard Wiredu, Okelue E Okobi, Peter Nebuwa, Esther I Ezeani, Amaka S Alozie

**Affiliations:** 1 Medicine, VN Karazin Kharkiv National University, Kharkiv, UKR; 2 Medicine, Saint James School of Medicine, Arnos Vale, VCT; 3 Family Medicine, IMG Research Academy &amp; Consulting LLC, Homestead, USA; 4 Internal Medicine, College of Health Sciences, Nnamdi Azikwe University, Nnewi, NGA; 5 Family Medicine, Indiana Regional Medical Center (IRMC), Indiana, USA; 6 Primary Care, Lifebridge Health, Baltimore, USA; 7 Medicine, Abia State University, Uturu, NGA

**Keywords:** artificial intelligence in healthcare, collaborative approach, deprescribing, digital health education, medication adherence, medication management, multimorbidity management, older adult, pharmacogenomics, polypharmacy

## Abstract

As the global population ages, polypharmacy-the concurrent use of multiple medications-has become increasingly common among older adults with multiple chronic conditions. While often clinically necessary, polypharmacy is associated with significant challenges, including medication nonadherence, adverse drug events, and increased hospitalizations. This narrative review synthesizes current evidence to explore the burden of polypharmacy, identify barriers to safe medication use, and assess intervention strategies aimed at improving outcomes in older adults.

Drawing from diverse sources including clinical trials, observational studies, and policy reports, this review highlights the multifaceted nature of medication management in older adults. Contributing factors to nonadherence include cognitive decline, regimen complexity, low health literacy, and fragmented care. Interventions such as pharmacist-led medication reviews, deprescribing initiatives, motivational interviewing, and digital health tools have demonstrated potential in improving adherence and therapeutic safety. Emerging approaches-including pharmacogenomic-guided prescribing and AI-driven clinical decision support-also offer promising avenues for personalized care.

A comprehensive, multidisciplinary framework that incorporates patient-centered communication, integrated care models, and supportive policy infrastructure is essential for addressing the complexities of polypharmacy in older populations. Continued research and collaboration across healthcare disciplines will be critical to translating these strategies into routine practice and enhancing medication safety for this vulnerable demographic.

## Introduction and background

The global aging population has led to a dramatic rise in the burden of chronic diseases, resulting in an increased reliance on multiple medications-a phenomenon known as polypharmacy, commonly defined as the concurrent use of five or more medications [1]. According to the World Health Organization, the number of people aged 60 years and older is projected to reach 2.1 billion by 2050, up from 1 billion in 2020, with a large proportion living with chronic conditions requiring long-term pharmacotherapy [2]. A 2023 OECD report estimates that approximately 50.1% of adults aged 75 and over in high-income countries are prescribed five or more medications concurrently, highlighting the widespread nature of polypharmacy and the need for appropriate medication management [3]. Among adults aged 65 years and older, the prevalence of polypharmacy ranges from 30% to 60%, and this trend is expected to grow as life expectancy continues to increase [4]. While the use of multiple medications can be necessary for managing complex comorbidities, it also poses significant risks. These include adverse drug events (ADEs), hospitalizations, medication errors, drug-drug interactions, cognitive decline, and poor treatment adherence [5].

Studies estimate that up to 50% of patients with chronic conditions do not adhere to prescribed medication regimens, contributing to increased morbidity, hospital admissions, and healthcare expenditures [6]. Medication adherence is influenced by a complex interplay of factors, including polypharmacy-related regimen complexity, side effects, mental health conditions, low health literacy, inadequate caregiver support, and fragmented healthcare delivery systems [7]. The transition of older adults between care settings further exacerbates medication discrepancies, often leading to therapeutic duplication or omission [8].

Current clinical guidelines are frequently designed for single-disease management and fail to accommodate the needs of patients with multimorbidity, thereby complicating the task of optimizing medication therapy for older adults [9]. For example, the American Diabetes Association’s (ADA's) Standards of Medical Care in Diabetes-2022 offer detailed glycemic control algorithms but provide limited guidance for tailoring treatment in the context of multimorbidity, such as coexisting heart failure or cognitive impairment [10]. The 2022 ADA guidelines acknowledge this limitation, particularly in the context of individualized care for older adults with multiple comorbidities [10]. This siloed approach can lead to therapeutic conflicts, overtreatment, and increased risk of adverse drug events in older patients managing multiple chronic conditions [9]. Inadequate integration of geriatric-specific pharmacological principles into routine care contributes to overprescribing, underprescribing, or the use of potentially inappropriate medications [7]. In response to these issues, a range of interventions has emerged, including pharmacist-led medication reviews [11], deprescribing initiatives [12], digital adherence tools [13], and pharmacogenomic-based prescribing strategies [14]. Although these approaches have demonstrated potential in improving medication outcomes, their implementation across diverse healthcare settings remains inconsistent [7].

This paper aims to synthesize current literature on the burden of polypharmacy and medication nonadherence in older adults, evaluate the effectiveness of intervention strategies, and propose a multidisciplinary model for optimizing medication management in this vulnerable population. The synthesis draws from both qualitative and quantitative studies, with an emphasis on peer-reviewed research that provides empirical evidence (such as clinical trials, cohort studies, and meta-analyses) as well as qualitative investigations that explore patient experiences, provider perspectives, and contextual barriers to adherence.

**Figure 1:**
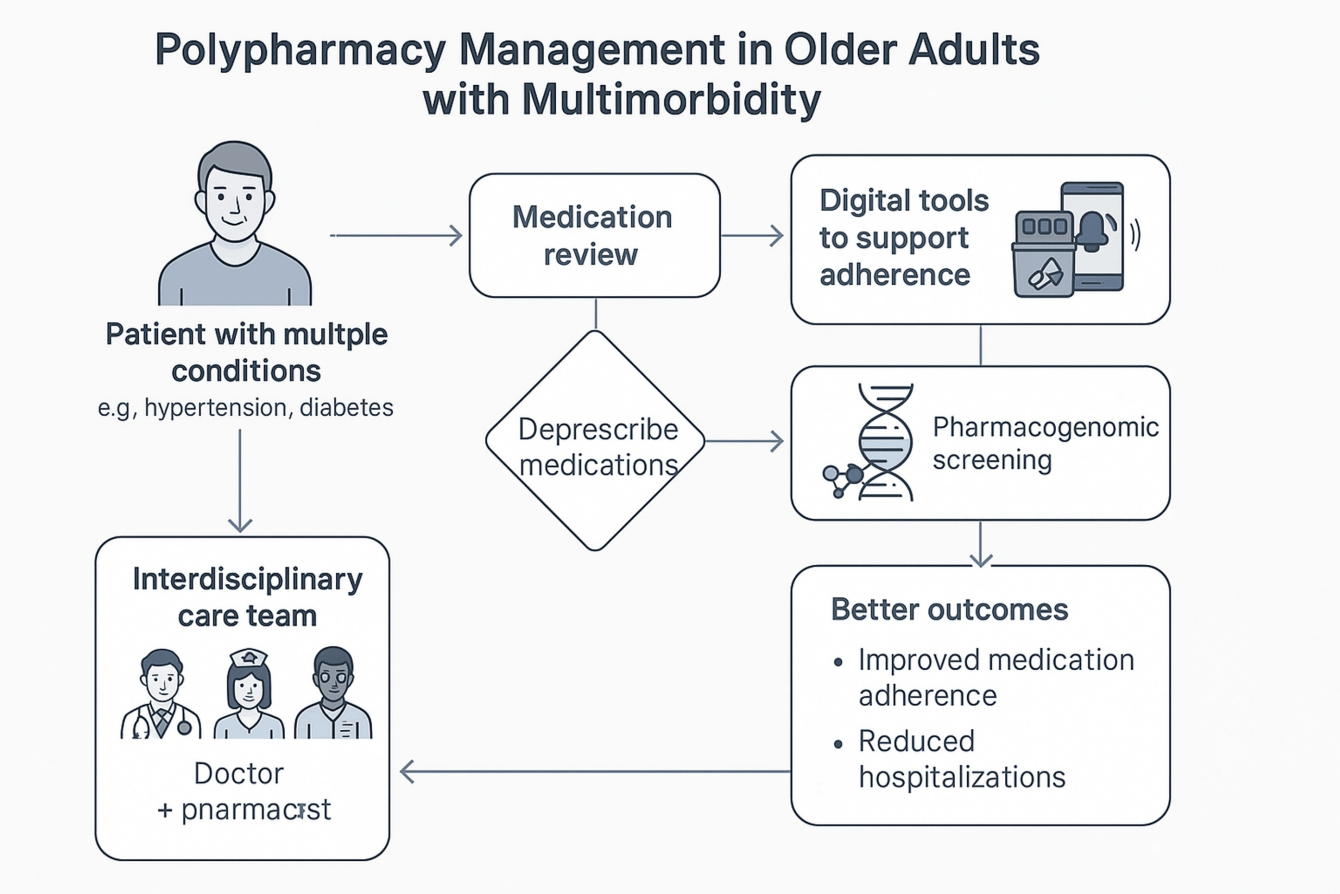
Polypharmacy management framework A visual summary of patient-centered strategies to manage polypharmacy in older adults with multimorbidity. Created by the author for this publication. No external sources were used.

To address this gap, Figure 1 presents an integrated, patient-centered framework for managing polypharmacy in older adults. The flowchart emphasizes key evidence-based strategies including regular medication reviews, deprescribing protocols, the use of digital adherence tools, and pharmacogenomic personalization. These interventions are supported by coordinated interdisciplinary collaboration to ensure safe, effective, and individualized medication management for older adults with multimorbidity.

## Review

Background and scope of the problem

As global life expectancy steadily increases, the prevalence of chronic, age-associated diseases such as hypertension, diabetes, osteoarthritis, and heart failure is rising in parallel. This has led to an unprecedented expansion in the use of multiple medications among older adults, which is a phenomenon known as polypharmacy, commonly defined as the concurrent use of five or more prescription drugs. In high-income countries, the prevalence of polypharmacy among individuals aged 65 years and above ranges from 30% to 60%, with projections indicating continued growth as the global population ages and clinical guidelines become more treatment-intensive in the context of multimorbidity [5].

Although polypharmacy may be clinically necessary for the management of complex comorbidities, it also introduces a wide array of risks. ADEs, medication errors, drug-drug and drug-disease interactions, cognitive impairment, frailty, and functional decline are well-documented complications associated with excessive or inappropriate medication use in the elderly. For instance, a meta-analysis by Fried et al. showed that polypharmacy is strongly associated with increased hospitalization, falls, and mortality in community-dwelling older adults [15]. Inappropriate polypharmacy, where the risks of certain medications outweigh their benefits, remains a significant contributor to medication-related harm, particularly when not guided by age-appropriate prescribing frameworks.

One of the most pressing consequences of polypharmacy is suboptimal medication adherence. It is estimated that approximately 50% of older adults with chronic illnesses fail to take their medications as prescribed, which leads to therapeutic failure, disease progression, preventable hospital admissions, and increased mortality [16]. Factors influencing nonadherence are multifactorial and include regimen complexity, polypharmacy-induced confusion, adverse side effects, depression, cognitive dysfunction, limited health literacy, financial constraints, and insufficient caregiver or social support [7]. Additionally, transitions of care (such as hospital discharge or transfer to skilled nursing facilities) are high-risk periods for medication discrepancies and discontinuities, often resulting in therapeutic duplication or omission [8].

The inadequacy of current clinical guidelines in addressing the needs of patients with multimorbidity further exacerbates the problem. Most existing guidelines are structured around the management of single conditions, offering limited guidance on prioritization or deprescribing in the context of multiple chronic illnesses. A landmark review by Boyd et al. highlighted how adherence to disease-specific guidelines in older adults with coexisting conditions can lead to medication overload, fragmented care, and avoidable harm [9]. This disconnect creates a dilemma for healthcare providers, who are expected to reconcile multiple protocols without a comprehensive, patient-centered prescribing framework.

Thus, a nuanced understanding of the scope of polypharmacy and its systemic, clinical, and behavioral determinants is essential to improve medication safety in older adults. Beyond merely counting medications, high-quality management of polypharmacy necessitates a shift toward integrated, individualized care models. These must incorporate multidisciplinary collaboration, geriatric pharmacology principles, routine medication reviews, and deprescribing where appropriate. Only by addressing the root causes of inappropriate prescribing and nonadherence can healthcare systems hope to reduce medication-related harm and optimize outcomes for aging populations.

Methods

This study employed a narrative review methodology to synthesize and critically evaluate the existing body of literature related to polypharmacy, medication adherence, and intervention strategies for older adults with multimorbidity. A narrative review was deemed appropriate given the heterogeneity in study designs, outcome measures, and interventions, as well as the need to contextualize multifaceted healthcare challenges within a comprehensive and interdisciplinary framework.

The search included peer-reviewed journal articles, systematic reviews, meta-analyses, clinical guidelines, observational studies (including cohort and case-control designs), and expert position statements published between January 2006 and April 2024. A total of 712 records were identified through database searches (PubMed, Scopus, CINAHL, and Google Scholar). After removing 126 duplicates, 586 titles and abstracts were screened. Of these, 402 articles were excluded for not meeting relevance or population criteria. The full texts of 184 articles were assessed for eligibility, and following full-text screening, 74 studies met the inclusion criteria and were synthesized in the review. These comprised 22 observational studies (cohort and case-control), 18 randomized controlled trials or quasi-experimental designs, 12 systematic reviews and meta-analyses, 10 qualitative studies, 6 clinical guidelines or expert consensus statements, and 6 mixed-methods or policy-focused publications. The search strategy combined Boolean operators and Medical Subject Headings (MeSH) aligned with the following keywords: polypharmacy, older adults, geriatric pharmacology, medication adherence, deprescribing, multimorbidity, pharmacogenomics, clinical decision support systems, digital health, care coordination, and medication reconciliation.

Inclusion Criteria

The following are the inclusion criteria: (i) studies focusing on individuals aged 60 years and above; (ii) interventions targeting polypharmacy reduction, medication adherence improvement, or deprescribing; (iii) research conducted in clinical, outpatient, or community-based healthcare settings; (iv) English-language publications with full-text access.

Exclusion Criteria

The following are the exclusion criteria: (i) studies exclusive to pediatric, palliative, or oncology-specific pharmacotherapy; (ii) non-peer-reviewed literature (e.g., editorials, commentaries, or press articles); (iii) studies with insufficient methodological transparency or that failed to report adherence-related outcomes.

Following deduplication, all articles were screened by title and abstract, followed by full-text review. Two independent reviewers conducted a methodological quality appraisal using tools adapted from the Preferred Reporting Items for Systematic Reviews and Meta-Analyses (PRISMA) and Critical Appraisal Skills Programme (CASP) frameworks to enhance rigor and minimize selection bias [17,18]. This dual-assessment protocol aligns with best practices in digital health and clinical pharmacology reviews targeting high-risk patient populations [19].

The data extracted from selected studies were organized using thematic synthesis, allowing categorization into three overarching domains: (i) epidemiology and clinical consequences of polypharmacy and nonadherence; (ii) intervention strategies (e.g., pharmacist-led reviews, digital adherence technologies, pharmacogenomics, and AI-driven decision support); (iii) implementation barriers and facilitators across diverse health systems.

A descriptive synthesis was conducted to evaluate and compare study outcomes, including changes in medication adherence rates, reduction in adverse drug events, hospitalization frequency, patient quality of life, and cost-effectiveness of interventions. Wherever applicable, meta-analytic or high-quality cohort data were prioritized to anchor the narrative in robust empirical evidence. This structured analytical approach was designed to support the development of patient-centered, scalable recommendations tailored to the geriatric population.

Table 1 summarizes selected peer-reviewed studies on interventions aimed at managing polypharmacy among older adults.

**Table 1:** Summary of key studies on polypharmacy interventions in older adults (2015–2021) This table [20-23] presents a summary of selected peer-reviewed studies on interventions aimed at managing polypharmacy among older adults. Each entry includes the author(s), year of publication, study design, population details, intervention type, and primary outcomes such as changes in medication adherence, adverse drug events (ADEs), hospitalization frequency, and quality of life. Arrows denote the direction of outcomes: ↑ indicates improvement/increase; ↓ indicates reduction/decrease. Studies were chosen based on methodological rigor and relevance to older populations with multimorbidity, in alignment with the objectives of this narrative review.

Author (year)	Study design	Sample size/population	Intervention type	Key outcomes
Marcum et al. (2021) [20]	Randomized controlled trial (RCT)	Elderly patients with polypharmacy	Pharmacist-led medication review	↑ Adherence, ↓ ADEs, ↓ Hospitalizations
Patel et al. (2015) [21]	Cohort/intervention study	Older adults	Electronic reminder systems	↑ Adherence; hospitalization impact not significant
Ibrahim et al. (2021) [22]	Systematic review	Frail older adults	Deprescribing interventions	Reduced medication burden
Bieri et al. (2021) [23]	Qualitative study	Home-dwelling older adults and caregivers	Belief-oriented interventions	Improved perceptions of polypharmacy

Discussion

The increasing global prevalence of polypharmacy among older adults is a reflection of the broader demographic trend of aging populations and the rising chronic disease burden. While the use of multiple medications is often clinically warranted, it simultaneously introduces substantial risks, including adverse drug events (ADEs), therapeutic duplication, drug-drug interactions, and increased rates of hospitalization and mortality. The consequences of polypharmacy extend beyond physiological effects, often impairing functional status, cognition, and overall quality of life. According to a large population-based cohort study in Sweden, individuals aged ≥75 using ten or more medications had a significantly increased risk of hospitalization and mortality compared to those on fewer medications [4].

Medication nonadherence is a critical contributor to poor health outcomes in this group. A meta-analysis of 51 studies involving older adults with chronic conditions reported an average nonadherence rate of 43%, with complex regimens and cognitive impairment cited as predominant barriers [24]. Tailored interventions that reduce regimen complexity, enhance patient education, and foster better communication between providers and patients have shown promise in improving adherence. For example, the use of fixed-dose combination pills and synchronized refill systems has been associated with improved patient satisfaction and reduced error rates [25].

Multidisciplinary care models are among the most effective approaches to managing polypharmacy. A randomized controlled trial conducted in Ireland (SPPiRE study) showed that general practitioner-led medication reviews incorporating patient priorities led to a significant reduction in medication count among older adults with multimorbidity, although changes in potentially inappropriate prescribing were limited [26]. Similarly, the integration of geriatricians into primary care settings has been associated with reductions in polypharmacy-related hospital admissions [27]. These models are most successful when embedded into systems that support ongoing medication review, shared decision-making, and deprescribing practices.

Technological innovations also offer promising avenues for intervention.

In recent years, smart medication adherence tools (such as sensor-enabled blister packs and mobile health applications) have emerged as promising innovations to support medication management in older adults, particularly those with cognitive impairment. A usability study involving older adults and smart blister packaging demonstrated a 28% improvement in adherence rates over a three-month period [28], underscoring the potential of these technologies to mitigate nonadherence. However, despite these promising outcomes, their effectiveness may be limited by the persistent digital divide-notably disparities in access to devices, internet connectivity, and digital literacy. This challenge is especially acute among older adults from rural, low-income, or minority populations, who may face compounded barriers such as limited technology exposure, sensory or motor impairments, and mistrust of digital interventions.

Drawing from the Unified Theory of Acceptance and Use of Technology (UTAUT), factors such as performance expectancy, effort expectancy, and facilitating conditions significantly influence user acceptance of health technologies in older adults [29]. In many cases, low perceived usefulness, interface complexity, and lack of caregiver support reduce sustained engagement. Therefore, successful implementation of adherence technologies must go beyond device provision, incorporating user-centered design, tailored education, and caregiver or community involvement to ensure that these tools are accessible, acceptable, and impactful across diverse older adult populations [29].

Pharmacogenomics is an emerging field that holds significant potential to transform prescribing by accounting for genetic variability in drug metabolism, particularly in older adults with polypharmacy. This approach has already demonstrated clinical utility in psychiatry, cardiology, and oncology, where pharmacogenomic-guided treatment improves efficacy and reduces adverse effects [30]. In geriatric populations, this is especially relevant due to the heightened risk of adverse drug reactions, drug-gene interactions, and treatment failure.

Several studies have validated the clinical and economic benefits of pharmacogenomics in older adults. For example, in a randomized controlled trial involving elderly home health patients with polypharmacy, pharmacogenomic-guided care led to a 39% reduction in hospitalizations and emergency visits over 60 days, compared to standard medication management [13]. Furthermore, a 2022 cost-effectiveness analysis by Morris et al. found that implementing multi-gene pharmacogenomic testing in adults aged 65 and older taking antidepressants or statins resulted in a cost per quality-adjusted life year (QALY) well below commonly accepted thresholds, particularly when testing results were reused for multiple drug decisions over time [31].

Despite these promising outcomes, key implementation challenges persist, including the integration of pharmacogenomic data into electronic health records (EHRs), the lack of clinical decision support tools in many systems, limited reimbursement models, and variability in testing standardization across laboratories. Addressing these barriers is essential to scaling the benefits of precision prescribing to older adult populations in real-world settings.

AI-based tools have shown promising results in optimizing geriatric pharmacotherapy. In one comparative study, AI-driven clinical decision support systems outperformed human pharmacists by 22% in identifying potentially inappropriate prescriptions among older adults, demonstrating higher sensitivity and specificity in flagging drug-drug and drug-disease interactions. However, the study was conducted in a simulated clinical environment using retrospective prescription data, which may not fully capture the complexity of real-time clinical decision-making [32]. Additionally, while AI tools excelled at pattern recognition and guideline-based alerts, they lacked contextual judgment and individualized nuance, which remain critical strengths of experienced pharmacists. These findings underscore the potential of AI as a complementary tool rather than a replacement, particularly when integrated into multidisciplinary medication review processes. Despite their potential, these systems require rigorous validation, clinician training, and ethical oversight to avoid unintended consequences.

Taken together, these findings emphasize the urgency of implementing coordinated, patient-centered approaches to polypharmacy management. This includes not only clinical and technological innovations, but also policy-level support for deprescribing frameworks, training in geriatric pharmacology, and the development of evidence-based guidelines that reflect the realities of multimorbidity. A robust response to polypharmacy must be holistic-one that integrates patient preferences, minimizes therapeutic burden, and ensures the safe, effective use of medications across the continuum of care.

To address barriers to adherence, several studies have proposed tailored interventions aligned with patient-specific needs. These are summarized in Table 2, which outlines practical strategies to mitigate common challenges such as regimen complexity and cognitive impairment.

**Table 2:** Barriers to medication adherence in older adults with polypharmacy and evidence-based strategies for improvement This table outlines key challenges contributing to medication nonadherence in older adults including regimen complexity, cognitive decline, low health literacy, and poor coordination of care. It also presents targeted strategies such as simplified dosing, caregiver support tools, patient education, deprescribing initiatives, and interdisciplinary collaboration. Adapted from Demonceau et al. and Pratiwi et al. [33,34].

Barrier	Recommended strategy
Complex regimens	Simplify dosing schedules; synchronize medications
Cognitive decline	Use smart packaging and caregiver reminders
Low health literacy	Tailored patient education
Side effects	Deprescribing and dose adjustments
Poor communication	Team-based care and follow-up

Recommendations

Addressing polypharmacy and medication nonadherence in older adults requires a multifaceted strategy that incorporates clinical, technological, and systemic interventions tailored to this vulnerable population.

One of the most effective and evidence-based strategies for managing polypharmacy in older adults is the routine implementation of comprehensive, pharmacist-led medication reviews. These interventions systematically evaluate medication appropriateness, identify potentially inappropriate prescriptions, and support deprescribing when warranted. A pivotal example is the OPTIMIZE trial, a cluster-randomized controlled study involving older adults with polypharmacy and cognitive impairment, which demonstrated that pharmacist-led deprescribing significantly reduced the number of medications without compromising symptom control or quality of life [35]. Additionally, a meta-analysis by Reeve et al. affirmed that pharmacist-led deprescribing interventions consistently reduce the use of potentially inappropriate medications and improve medication safety across various care settings [36]. Collectively, this body of evidence underscores the clinical value of involving pharmacists in medication optimization to enhance adherence, reduce adverse drug events, and improve patient-centered outcomes in geriatric populations.

The integration of digital adherence tools (such as smart pill bottles, mobile health apps, and reminder systems) has shown promise in enhancing medication-taking behaviors, particularly among older adults with mild cognitive impairment or low health literacy [37]. These tools not only improve adherence but also enable real-time monitoring and early detection of nonadherence trends that clinicians can act upon.

Pharmacogenomics presents another promising frontier. By tailoring drug regimens based on genetic variability in drug metabolism, clinicians can minimize adverse drug reactions and optimize drug efficacy. Although adoption is still limited due to cost and infrastructure barriers, studies suggest that pharmacogenomic testing significantly improves patient outcomes when integrated into routine care for older adults with polypharmacy [13].

To support sustainable medication management, interdisciplinary collaboration is essential. This includes coordination between physicians, pharmacists, nurses, caregivers, and, where applicable, community health workers. Such collaboration ensures that interventions are patient-centered, feasible, and aligned with functional goals and life expectancy [38].

Finally, systemic changes are needed. Policymakers and healthcare organizations should incorporate polypharmacy management standards into clinical guidelines, electronic health records (EHRs), and quality assurance frameworks. Education and training programs for clinicians should also emphasize deprescribing principles, geriatric pharmacology, and shared decision-making strategies. While frameworks such as the World Health Organization’s Medication Without Harm initiative [39] and the American Geriatrics Society Beers Criteria [40] provide guidance on identifying potentially inappropriate medications, integration into clinical workflows remains inconsistent. Additionally, few national healthcare systems have formally embedded deprescribing protocols into reimbursement models, clinical performance metrics, or electronic decision support systems, creating a significant gap between evidence and practice [41,42]. Addressing these gaps requires multilevel policy interventions, including regulatory endorsement, provider incentives, and cross-disciplinary training to ensure that safe and appropriate medication use becomes a routine aspect of geriatric care.

## Conclusions

In summary, addressing the complex issues of polypharmacy and medication nonadherence in older adults demands a comprehensive and person-centered approach-one that acknowledges the full spectrum of their physical, emotional, and social needs. The success of initiatives such as pharmacist-led reviews, deprescribing strategies, and digital adherence tools relies heavily on strong interdisciplinary collaboration, meaningful patient engagement, and supportive healthcare systems that promote continuity of care. Emerging innovations like pharmacogenomic testing and AI-driven clinical decision support hold great promise for personalizing treatment, but their integration must be thoughtful, ethically sound, and anchored in compassionate clinical practice. Lasting progress will depend on empowering the people at the heart of care. Advancing safer, more effective medication use for older adults calls for coordinated efforts across providers, caregivers, health systems, and policymakers alike.
